# Taxonomic reassessment and phylogenetic placement of *Cyrtodactylus phuketensis* (Reptilia, Gekkonidae) based on morphological and molecular evidence

**DOI:** 10.3897/zookeys.1040.65750

**Published:** 2021-05-28

**Authors:** Korkhwan Termprayoon, Attapol Rujirawan, L. Lee Grismer, Perry L. Wood Jr., Anchalee Aowphol

**Affiliations:** 1 Department of Zoology, Faculty of Science, Kasetsart University, Bangkok, 10900, Thailand Kasetsart University Bangkok Thailand; 2 Herpetology Laboratory, Department of Biology, La Sierra University, 4500 Riverwalk Parkway, Riverside, California 92515, USA La Sierra University Riverside United States of America; 3 Department of Biological Sciences and Museum of Natural History, Auburn University, Auburn, AL, USA Auburn University Auburn United States of America

**Keywords:** *Cyrtodactylus macrotuberculatus*, Malaysia, morphology, phylogeny, taxonomic status, Thailand, Thai-Malay Peninsula

## Abstract

The taxonomy and phylogeny of the *Cyrtodactylus pulchellus* complex along the Thai-Malay Peninsular region has been the focus of many recent studies and has resulted in the recognition of 17 species. However, the majority of these studies were focused on Peninsular and insular Malaysia where there were specimens and genetic vouchers. The taxonomic status and phylogenetic relationships of the Thai species in this complex remain unresolved, due to the lack of genetic material of some species, especially *C. phuketensis* and *C. macrotuberculatus* from Thai populations. In this study, we investigated the phylogenetic relationship between *C. phuketensis* and its closely related species *C. macrotuberculatus*, using both morphometric and molecular data. Phylogenetic analyses of mitochondrial NADH dehydrogenase subunit 2 (ND2) gene revealed that *C. phuketensis* is embedded within a *C. macrotuberculatus* clade with 1.45–4.20% (mean 2.63%) uncorrected pairwise sequence divergences. Morphological comparisons showed nearly identical measurements of *C. phuketensis* and *C. macrotuberculatus* and overlapping ranges in meristic characters. Based on these data, *C. phuketensis* is considered to be a variant of *C. macrotuberculatus*, thus rendering *C. phuketensis* a junior synonym of *C. macrotuberculatus*.

## Introduction

*Cyrtodactylus* is a genus of the bent-toed geckos which is widely distributed across South Asia to Melanesia (Wood et al. 2012; Grismer et al. 2020, 2021a). This genus is the most speciose group of gekkotans, with 306 species currently recognized (Uetz et al. 2020). Due to discoveries of hidden taxa within species complexes over decades, species diversity of *Cyrtodactylus* has remarkably increased, especially in Southeast Asia (e.g., Grismer et al. 2012, 2018; Riyanto et al. 2017; Pauwels et al. 2018; Murdoch et al. 2019; Quah et al. 2019). In the last decade, the integrative approach of molecular and morphological data has been applied to study species boundaries, evaluate taxonomic status, and the adaptive evolution in habitat preference in *Cyrtodactylus* (Grismer et al. 2015, 2020, 2021a, b; Nielsen and Oliver 2017).

The *Cyrtodactylus pulchellus* group (Grismer et al. 2021a) is widely distributed along the Thai-Malay Peninsular region and extends from lowland to over 1,500 meters above sea level (Grismer 2011). It was recognized as a single species until morphological differences from an insular population (Pulau Langkawi, Kedah, Malaysia) were noticed and a new species, *C. macrotuberculatus* Grismer and Ahmad, 2008 was recognized. Several populations of *C. pulchellus* were considered to be part of a species complex that may reveal hidden diversity and unnamed species (Grismer 2011; Grismer et al. 2012). To date, taxonomic revisions of the *C. pulchellus* group have recovered 17 named species based on molecular and morphological data, including *C. astrum*Grismer et al., 2012, *C. australotitiwangsaensis*Grismer et al., 2012, *C. bintangrendah*Grismer et al., 2012, *C. bintangtinggi*Grismer et al., 2012, *C. dayangbuntingensis*Quah et al., 2019, *C. evanquahi* Wood et al., 2020, *C. hidupselamanya*Grismer et al., 2016, *C. jelawangensis*Grismer et al., 2014, *C. langkawiensis*Grismer et al., 2012, *C. lekaguli*Grismer et al., 2012, *C. lenggongensis*Grismer et al., 2016, *C. macrotuberculatus*, *C. pulchellus* Gray, 1827, *C. phuketensis*Sumontha et al., 2012 (only morphological data provided), *C. sharkari*Grismer et al., 2014, *C. timur*Grismer et al., 2014, and *C. trilatofasciatus*Grismer et al., 2012. Among this complex group, *C. macrotuberculatus* and *C. phuketensis* showed minor morphological differences based on the original description (Sumontha et al. 2012) but no genetic data were provided for elucidating their phylogenetic placement within the *C. pulchellus* group.

*Cyrtodactylus phuketensis* was described as a new species from Ban Bangrong, Thalang District, Phuket Province by Sumontha et al. (2012). It was similar to *C. macrotuberculatus* in having tuberculation on ventral surface of the forelimbs, gular region and ventrolateral folds, and relatively larger ventral scales (compared to other species in *C. pulchellus* complex). In the original description, *C. phuketensis* was separated from *C. macrotuberculatus* by having three dark bands between limb insertions, 19 subdigital lamellae on the 4^th^ toe, the presence of a precloacal groove in both sexes, and eight dark caudal bands on an original tail.

During our field surveys, nine specimens of *C. phuketensis* were collected from the type locality and nearby areas and we found variation in the number of body bands and overlap in the ranges of putatively diagnostic meristic characters when compared to *C. macrotuberculatus*. Therefore, this study aims to reassess the taxonomic status of *C. macrotuberculatus* and *C. phuketensis* using morphological and genetic data from the mitochondrial NADH dehydrogenase subunit 2 (ND2) gene and flanking tRNAs. The analyses were performed on newly collected specimens from southern Thailand and from the type specimens of both species.

## Materials and methods

### Specimen sampling

**Figure 1.**
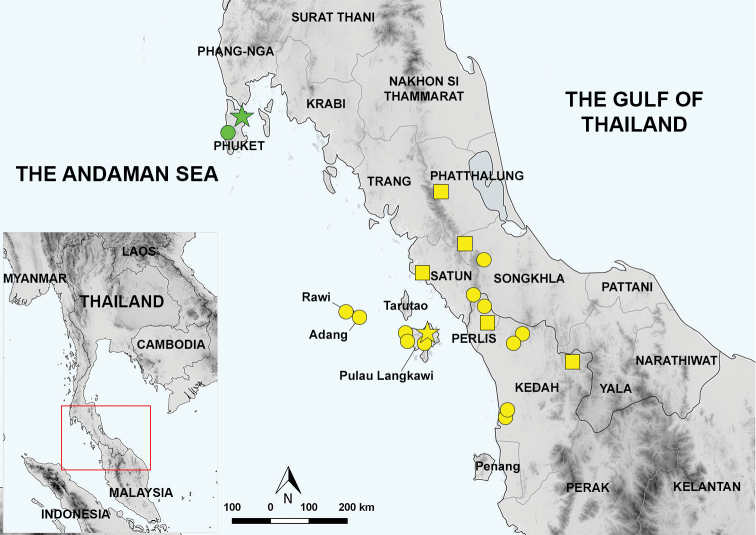
Map illustrating the known geographic distribution of *Cyrtodactylus macrotuberculatus* and *C. phuketensis*. Yellow star: the type locality of *C. macrotuberculatus* at Gunung Raya, Pulau Langkawi, Kedah, Malaysia. Green star: the type locality of *C. phuketensis* at Thalang District, Phuket Island, Phuket Province. Yellow circles: *C. macrotuberculatus* samples used in this study. Green circle: *C. phuketensis* samples used in this study. Yellow squares: the distribution of *C. macrotuberculatus* taken from Grismer et al. (2012), and Quah et al. (2019). The samples used correspond to those in Table 1.

During October 2017 and June 2019, field surveys were conducted at five localities in southern Thailand, including the type locality of *C. phuketensis* (Fig. 1; Table 1). Specimens were investigated and captured by hand during the night (1900–2300). Liver or muscle tissues were individually preserved in 95% ethyl alcohol and stored at -20 °C for molecular analysis. Specimens were fixed in 10% formalin and later transferred to 70% ethyl alcohol. Preserved specimens were deposited in the herpetological collections of the Zoological Museum, Kasetsart University, Thailand (**ZMKU**). Additional specimens were also examined from the Princess Maha Chakri Sirindhorn Natural History Museum (**PSU**), Prince of Songkhla University, Thailand; Thailand Natural History Museum (**THNHM**), Thailand; La Sierra University Herpetological Collection (**LSUHC**), La Sierra University, Riverside, California, USA; and the Zoological Reference Collection (**ZRC**) of Lee Kong Chian Natural History Museum at National University of Singapore, Singapore.

**Table 1.** Specimens of *Cyrtodactylus* used in (A) molecular and/or (B) morphological analyses in this study. WM = West Malaysia; TH = Thailand.

Species	Locality	Museum No.	GenBank Accession No.	Type of analysis	Reference
*Hemidactylus frenatus*	Unknow	NC 00155	JX519468	A	Grismer et al. (2012)
*Agamura persica*	Pakistan, Baluchistan Province, Makran District, Gwadar	FMNH 247474	JX440515	A	Grismer et al. (2012)
*Tropiocolotes steudneri*	Unknow	JB 28	JX440520	A	Grismer et al. (2012)
*Cyrtodactylus elok*	WM, Pahang, Fraser’s Hill, The Gap	LSUHC 6471	JQ889180	A	Grismer et al. (2012)
*C. intermedius*	TH, Chantaburi Province, Khao Khitchakut District	LSUHC 9513	JX519469	A	Grismer et al. (2012)
	TH, Chantaburi Province, Khao Khitchakut District	LSUHC 9514	JX519470	A	Grismer et al. (2012)
	Laos, Khammouan Province, Nakai District	FMNH 255454	JQ889181	A	Grismer et al. (2012)
*Cyrtodactylus* sp.	TH, Loei, Phu Rua	FMNH 265806	JX519471	A	Grismer et al. (2012)
*C. astrum*	WM, Perlis, Gua Kelam	LSUHC 8806	JX519481	A	Grismer et al. (2012)
	WM, Perlis, Gua Kelam	LSUHC 8808	JX519479	A	Grismer et al. (2012)
	WM, Perlis, Kuala Perlis	LSUHC 8815	JX519482	A	Grismer et al. (2012)
	WM, Perlis, Kuala Perlis	LSUHC 8816 (paratype)	JX519483	A	Grismer et al. (2012)
*C. australotitiwangsaensis*	WM, Pahang, Genting Highlands	LSUHC 6637 (holotype)	JX519484	A	Grismer et al. (2012)
	WM, Pahang, Fraser’s Hill	LSUHC 8086	JX519486	A	Grismer et al. (2012)
	WM, Pahang, Fraser’s Hill	LSUHC 8087	JX519485	A	Grismer et al. (2012)
*C. bintangrendah*	WM, Kedah, Bukit Palang	LSUHC 9984	JX519487	A	Grismer et al. (2012)
	WM, Kedah, Bukit Mertajam	LSUHC 10331 (paratype)	MN125076	A	Quah et al. (2019)
	WM, Kedah, Bukit Mertajam	LSUHC 10519	MN125077	A	Quah et al. (2019)
	WM, Kedah, Bukit Mertajam	LSUHC 10520 (paratype)	MN125078	A	Quah et al. (2019)
*C. bintangtinggi*	WM, Perak, Bukit Larut	LSUHC 8862	JX519493	A	Grismer et al. (2012)
	WM, Perak, Bukit Larut	LSUHC 9006 (paratype)	JX519494	A	Grismer et al. (2012)
*C. dayangbuntingensis*	WM, Kedah, Dayang Bunting Island	LSUHC 14353	MN125090	A	Quah et al. (2019)
	WM, Kedah, Dayang Bunting Island	LSUHC 14354	MN125091	A	Quah et al. (2019)
	WM, Kedah, Dayang Bunting Island	LSUHC 14355	MN125092	A	Quah et al. (2019)
*C. evanquahi*	WM, Kedah, Gunung Baling	BYU 53435 (holotype)	MN586889	A	Wood et al. (2020)
	WM, Kedah, Gunung Baling	BYU 53436 (paratype)	MN586890	A	Wood et al. (2020)
	WM, Kedah, Gunung Baling	BYU 53437 (paratype)	MN586891	A	Wood et al. (2020)
*C. hidupselamanya*	WM, Kelantan, Felda Chiku 7	LSUHC 12158 (paratype)	KX011412	A	Grismer et al. (2016)
	WM, Kelantan, Felda Chiku 7	LSUHC 12160 (paratype)	KX011414	A	Grismer et al. (2016)
	WM, Kelantan, Felda Chiku 7	LSUHC 12161 (paratype)	KX011415	A	Grismer et al. (2016)
	WM, Kelantan, Felda Chiku 7	LSUHC 12162 (paratype)	KX011416	A	Grismer et al. (2016)
	WM, Kelantan, Felda Chiku 7	LSUHC 12163 (holotype)	KX011417	A	Grismer et al. (2016)
	WM, Kelantan, Felda Chiku 7	LSUHC 12173 (paratype)	KX011420	A	Grismer et al. (2016)
*C. jelawangensis*	WM, Gunung Stong, Kelantan	LSUHC 11060 (paratype)	KJ659850	A	Grismer et al. (2014)
	WM, Kelantan, Gunung Stong	LSUHC 11061 (paratype)	KJ659851	A	Grismer et al. (2014)
	WM, Gunung Stong, Kelantan	LSUHC 11062 (holotype)	KJ659852	A	Grismer et al. (2014)
*C. langkawiensis*	WM, Kedah, Pulau Langkawi, Wat Wanaram	LSUHC 9123 (paratype)	JX519500	A	Grismer et al. (2012)
	WM, Kedah, Pulau Langkawi, Wat Wanaram	LSUHC 9124 (paratype)	JX519499	A	Grismer et al. (2012)
	WM, Kedah, Pulau Langkawi, Wat Wanaram	LSUHC 9125	JX519496	A	Grismer et al. (2012)
	WM, Kedah, Pulau Langkawi, Wat Wanaram	LSUHC 9435	JX519495	A	Grismer et al. (2012)
*C. lekaguli*	TH, Phang-nga Province, Takua Pa District	ZMKU 00720	KX011425	A	Grismer et al. (2016)
	TH, Phang-nga Province, Takua Pa District	ZMKU 00721	KX011426	A	Grismer et al. (2016)
	TH, Phang-nga Province, Takua Pa District	ZMKU 00722	KX011427	A	Grismer et al. (2016)
	TH, Phang-nga Province, Takua Pa District	ZMKU 00723	KX011428	A	Grismer et al. (2016)
*C. lenggongensis*	WM, Perak, Lenggong Valley	LSUHC 9974 (holotype)	JX519490	A	Grismer et al. (2012)
	WM, Perak, Lenggong Valley	LSUHC 9975 (paratype)	JX519488	A	Grismer et al. (2012)
	WM, Perak, Lenggong Valley	LSUHC 9976 (paratype)	JX519489	A	Grismer et al. (2012)
	WM, Perak, Lenggong Valley	LSUHC 9977 (paratype)	JX519491	A	Grismer et al. (2012)
*C. macrotuberculatus*	WM, Kedah, Kuala Nerang	BYU 51869	MN125085	A	Quah et al. (2019)
	WM, Kedah, Kuala Nerang	BYU 51870	MN125086	A	Quah et al. (2019)
	WM, Kedah, Gunung Jerai	LSUHC 5939	JX519513	A	Grismer et al. (2012)
	WM, Kedah, Gunung Jerai	LSUHC 5999	JX519512	A	Grismer et al. (2012)
	WM, Kedah, Gunung Jerai	LSUHC 6000	JX519514	A	Grismer et al. (2012)
	WM, Kedah, Pulau Langkawi, Lubuk Sembilang	LSUHC 6829	JX519505	A	Grismer et al. (2012)
	WM, Kedah, Pulau Langkawi, Gunung Machinchang	LSUHC 7560	JX519503	A	Grismer et al. (2012)
*C. macrotuberculatus*	WM, Kedah, Pulau Langkawi, Gunung Raya	LSUHC 9428	JX519506	A, B	Grismer et al. (2012), This study
	WM, Kedah, Pulau Langkawi, Gunung Raya	LSUHC 9429	–	B	This study
	WM, Kedah, Pulau Langkawi, Gunung Raya	LSUHC 9432	–	B	This study
	WM, Kedah, Pulau Langkawi, Gunung Machinchang	LSUHC 9448	JX519507	A	Grismer et al. (2012)
	WM, Kedah, Pulau Langkawi, Gunung Machinchang	LSUHC 9449	JX519509	A	Grismer et al. (2012)
	WM, Kedah, Hutan Lipur Sungai Tupah	LSUHC 9671	JX519510	A	Grismer et al. (2012)
	WM, Kedah, Hutan Lipur Sungai Tupah	LSUHC 9672	JX519511	A	Grismer et al. (2012)
	WM, Kedah, Hutan Lipur Sungai Tupah	LSUHC 9693	JX519517	A	Grismer et al. (2012)
	WM, Perlis, Perlis State Park	LSUHC 9980	JX519515	A	Grismer et al. (2012)
	WM, Perlis, Perlis State Park	LSUHC 9981	JX519516	A, B	Grismer et al. (2012), This study
	WM, Perlis, Bukit Chabang	LSUHC 10037	JX519519	A	Grismer et al. (2012)
	WM, Perlis, Bukit Chabang	LSUHC 10038	JX519518	A	Grismer et al. (2012)
	WM, Perlis, Perlis State Park	LSUHC 10067	–	B	This study
	WM, Kedah, Bukit Wang	LSUHC 10329	MN125088	A	Quah et al. (2019)
	WM, Kedah, Bukit Wang	LSUHC 10330	MN125087	A	Quah et al. (2019)
	WM, Perlis, Perlis State Park	ZRC 2.4869	–	B	This study
	WM, Kedah, Pulau Langkawi, Gunung Raya	ZRC 2.6754 (holotype)	–	B	This study
	WM, Kedah, Pulau Langkawi, Gunung Raya	ZRC 2.6755 (paratype)	–	B	This study
	WM, Kedah, Pulau Langkawi, Gunung Raya	ZRC 2.6756 (paratype)	–	B	This study
	WM, Kedah, Pulau Langkawi, Telaga Tujuh	ZRC 2.6757/ LSUHC 7173 (paratype)	JX519508	A	Grismer et al. (2012)
	WM, Kedah, Pulau Langkawi, Lubuk Semilang	ZRC 2.6758 (paratype)	–	B	This study
	TH, Satun Province, Mueang Satun District, Adang Island	ZMKU R 00871	–	B	This study
	TH, Satun Province, Mueang Satun District, Adang Island	ZMKU R 00872	–	B	This study
	TH, Satun Province, Mueang Satun District, Adang Island	ZMKU R 00873	–	B	This study
	TH, Satun Province, Mueang Satun District, Adang Island	ZMKU R 00874	MW809294	A, B	This study
	TH, Satun Province, Mueang Satun District, Adang Island	ZMKU R 00875	MW809295	A, B	This study
	TH, Songkhla Province, Hat Yai District, Thung Tam Sao	ZMKU R 00876	MW809296	A, B	This study
	TH, Songkhla Province, Hat Yai District, Thung Tam Sao	ZMKU R 00877	MW809297	A, B	This study
	TH, Songkhla Province, Hat Yai District, Thung Tam Sao	ZMKU R 00878	MW809298	A, B	This study
	TH, Satun Province, Mueang Satun District, Adang Island	ZMKU R 00879	–	B	This study
	TH, Satun Province, Mueang Satun District, Adang Island	ZMKU R 00880	–	B	This study
	TH, Satun Province, Mueang Satun District, Adang Island	ZMKU R 00881	–	B	This study
	TH, Satun Province, Mueang Satun District, Adang Island	ZMKU R 00882	–	B	This study
	TH, Satun Province, Mueang Satun District, Rawi Island	ZMKU R 00883	MW809299	A, B	This study
	TH, Satun Province, Mueang Satun District, Rawi Island	ZMKU R 00884	–	B	This study
	TH, Satun Province, Mueang Satun District, Rawi Island	ZMKU R 00885	–	B	This study
	TH, Satun Province, Mueang Satun District, Rawi Island	ZMKU R 00886	–	B	This study
	TH, Satun Province, Mueang Satun District, Rawi Island	ZMKU R 00887	MW809300	A, B	This study
	TH, Satun Province, Mueang Satun District, Rawi Island	ZMKU R 00888	–	B	This study
	TH, Satun Province, Mueang Satun District, Rawi Island	ZMKU R 00889	–	B	This study
*C. macrotuberculatus* (as *C. phuketensis*)	TH, Phuket Province, Kathu District, Kathu Waterfall	ZMKU R 00890	MW809301	A, B	This study
	TH, Phuket Province, Kathu District, Kathu Waterfall	ZMKU R 00891	MW809302	A, B	This study
	TH, Phuket Province, Kathu District, Kathu Waterfall	ZMKU R 00892	MW809303	A, B	This study
	TH, Phuket Province, Kathu District, Kathu Waterfall	ZMKU R 00893	MW809304	A, B	This study
	TH, Phuket Province, Thalang District, Thep Krasatti	ZMKU R 00894	MW809305	A, B	This study
	TH, Phuket Province, Thalang District, Thep Krasatti	ZMKU R 00895	MW809306	A, B	This study
	TH, Phuket Province, Thalang District, Thep Krasatti	ZMKU R 00896	MW809307	A, B	This study
	TH, Phuket Province, Kathu District, Kathu Waterfall	ZMKU R 00897	MW809308	A, B	This study
	TH, Phuket Province, Kathu District, Kathu Waterfall	ZMKU R 00898	MW809309	A	This study
	TH, Phuket Province, Thalang District, Thep Krasatti	PSUZC-RT 2010.58	–	B	This study
	TH, Phuket Province, Thalang District, Thep Krasatti	THNHM 15378	–	B	This study
*C. pulchellus*	WM, Penang, Pulau Pinang, Empangan Air Itam	LSUHC 6668	JX519523	A	Grismer et al. (2012)
	WM, Penang, Pulau Pinang, Moongate Trail	LSUHC 6726	JX519527	A	Grismer et al. (2012)
	WM, Penang, Pulau Pinang, Moongate Trail	LSUHC 6727	JX519526	A, B	Grismer et al. (2012), This study
	WM, Penang, Pulau Pinang, Moongate Trail	LSUHC 6728	JX519525	A, B	Grismer et al. (2012), This study
	WM, Penang, Pulau Pinang, Moongate Trail	LSUHC 6729	JX519528	A, B	Grismer et al. (2012), This study
	WM, Penang, Pulau Pinang, Moongate Trail	LSUHC 6785	JX519524	A	Grismer et al. (2012)
	WM, Penang, Pulau Pinang, Air Terjun Titi Kerawang	LSUHC 9667	JX519520	A	Grismer et al. (2012)
	WM, Penang, Pulau Pinang, Air Terjun Titi Kerawang	LSUHC 9668	JX519521	A	Grismer et al. (2012)
	WM, Penang, Pulau Pinang, Air Terjun Titi Kerawang	LSUHC 10022	JX519522	A, B	Grismer et al. (2012), This study
	WM, Penang, Pulau Pinang	ZRC 2.4854	–	B	This study
	WM, Penang, Pulau Pinang	ZRC 2.4857	–	B	This study
*C. sharkari*	WM, Pahang, Merapoh, Gua Gunting	LSUHC 11022 (holotype)	KJ659853	A	Grismer et al. (2014)
*C. timur*	WM, Gunung Tebu, Terengganu	LSUHC 10886	KJ659854	A	Grismer et al. (2014)
	WM, Gunung Tebu, Terengganu	LSUHC 11183 (paratype)	KJ659855	A	Grismer et al. (2014)
	WM, Gunung Tebu, Terengganu	LSUHC 11184 (paratype)	KJ659856	A	Grismer et al. (2014)
	WM, Gunung Tebu, Terengganu	LSUHC 11185 (paratype)	KJ659857	A	Grismer et al. (2014)
*C. trilatofasciatus*	WM, Pahang, Cameron Highlands	LSUHC 10064	JX519529	A	Grismer et al. (2012)
	WM, Pahang, Cameron Highlands	LSUHC 10065	JX519530	A	Grismer et al. (2012)
	WM, Pahang, Cameron Highlands	LSUHC 10066	JX519531	A	Grismer et al. (2012)

### Molecular analyses

Total genomic DNA was extracted from 95% ethanol-preserved muscle or liver tissue using a NucleoSpin Tissue Kit (Macherey-Nagel GmbH & Co. KG, Germany). Mitochondrial NADH dehydrogenase subunit 2 (ND2) gene and flanking tRNAs were amplified via double-stand Polymerase Chain Reaction (PCR) using primers L4437a (tRNAmet: 5’ AAGCTTTCGGGCCCATACC 3’) and H5934 (COI: 5’ AGRGTGCCAATGTCTTTGTGRTT 3’) (Macey et al. 1997). PCR amplification occurred with an initial denaturation at 94 °C for 4 min, followed by 35 cycles of denaturation at 94 °C for 30 sec, annealing at 48–52 °C for 30 sec, extension at 72 °C for 1 min 30 sec, and final extension at 72 °C for 7 min. Amplification products were purified using NucleoSpin Gel and PCR Clean-Up kit (Macherey-Nagel GmbH & Co. KG, Germany) and visualized on 1.0% agarose gel electrophoresis. Purified PCR products were sequenced in both directions using amplifying primers on an ABI 3730XL DNA Sequencer (Applied Biosystems, CA, USA). Sequences were manually edited and aligned in Geneious R11 (Biomatters, Ltd, Auckland, New Zealand). The ND2 nucleotide sequences were translated to amino acid for the protein-coding region and to ensure the lack of stop codons. All sequences were deposited in GenBank under the accession numbers MW809294 to MW809309 (Table 1).

Phylogenetic relationships were inferred using two model based approaches, Bayesian Inference (BI) and Maximum Likelihood (ML). Outgroup species used to root the tree were *Hemidactylus frenatus*, *Agamura persica*, *Tropiocolotes steudneri*, *C. elok*, *C. intermedius*, *C. interdigitalis*, and *Cyrtodactylus* sp. based on Wood et al. (2012). The best-fit nucleotide substitution model for each of the three codon partitions and tRNAs was selected under the Bayesian Information Criterion (BIC) in PartitionFinder2 on XSEDE (Lanfear et al. 2016) using CIPRES (Cyberinfrastructure for Phylogenetic Research; Miller et al. 2010). BI analysis was executed in MrBayes 3.2.6 on XSEDE (Ronquist et al. 2012) using CIPRES with the TRN+I+G for the 1^st^ and 2^nd^ codon position, and TIM+I+G for the 3^rd^ codon position and tRNAs. Four chains (three hot and one cold) were run for 10,000,000 generations and sampled every 1,000 generations using Markov chain Monte Carlo (MCMC). To build a consensus tree, we discarded the first 25% of each run as burn-in and assessed stationarity by plotting log-likelihood score in Tracer ver. 1.7.1. (Rambaut et al. 2018). The ML analysis was performed on the web server W-IQ-TREE (Trifinopoulos et al. 2016) with 1,000 bootstrap replicates using ultrafast bootstrap approximation (Minh et al. 2013). Nodes having Bayesian posterior probabilities (BPP) of ≥ 0.95 and ultrafast bootstrap support (UFB) of ≥ 95 were considered to be strongly supported (Huelsenbeck and Ronquist 2001; Wilcox et al. 2002; Minh et al. 2013). Uncorrected pairwise sequence divergence was calculated to assess within and among species differences using the default settings in MEGA X 10.0.5 (Kumar et al. 2018).

### Morphological measurements

Morphological and meristic characters were modified from the previous studies of Grismer and Ahmad (2008) and Grismer et al. (2012). Measurements were taken with digital calipers to the nearest 0.1 mm for the following sixteen characters:

**SVL** snout-vent length, taken from the tip of snout to the vent;

**TW** tail width, taken at the base of the tail immediately posterior to the postcloacal swelling;

**TL** tail length, taken from vent to the tip of the tail, original or regenerated;

**FL** forearm length, taken from the posterior margin of the elbow while flexed 90° to the inflection of the flexed wrist;

**TBL** tibia length, taken from the posterior surface of the knee while flexed 90° to the base of the heel;

**AG** axilla to groin length, taken from the posterior margin of the forelimb at its insertion point on the body to the anterior margin of the hind limb at its insertion point on the body;

**HL** head length, the distance from the posterior margin of the retroarticular process of the lower jaw to the tip of the snout;

**HW** head width, measured at the angle of the jaws;

**HD** head depth, the maximum height of head from the occiput to the throat;

**ED** eye diameter, the greatest horizontal diameter of the eye-ball;

**EE** eye to ear distance, measured from the anterior edge of the ear opening to the posterior edge of the eye-ball;

**ES** eye to snout distance, measured from anterior most margin of the eye-ball to the tip of snout;

**EN** eye to nostril distance, measured from the anterior margin of the eye-ball to the posterior margin of the external nares;

**IO** inter orbital distance, measured between the anterior edges of the orbit;

**EL** ear length, the greatest vertical distance of the ear opening;

**IN** internarial distance, measured between the nares across the rostrum.

Additional scale counts and non-meristic characters evaluated were the number of supralabial and infralabial scales counted from the largest scale immediately posterior to the dorsal inflection of the posterior portion of the upper jaw to the rostral and mental scales, respectively; the number of paravertebral tubercles between limb insertions counted in a straight line immediately left of the vertebral column; the number of longitudinal rows of body tubercles counted transversely across the center of the dorsum from one ventrolateral fold to the other; the number of longitudinal rows of ventral scales counted transversely across the center of the abdomen from one ventrolateral fold to the other; the presence or absence of tubercles on the ventral surface of the forearm; the presence or absence of tubercles in the gular region, throat, and lateral margins of the abdomen; the number of subdigital lamellae beneath the fourth toe counted from the base of the first phalanx to the claw; the total number of precloacal and femoral pores (i.e., the contiguous rows of femoral and precloacal scales bearing pores combined as a single meristic referred to as the femoroprecloacal pores); the presence or absence of a precloacal depression or groove; the degree of body tuberculation, weak tuberculation referring to dorsal body tubercles that are low and rounded whereas prominent tuberculation refers to tubercles that are raised and keeled; the width of the dark body bands relative to the width of the interspace between the bands; number of dark caudal bands on the original tail; the white caudal bands of adults immaculate or infused with dark pigment; and whether or not the posterior portion of the original tail in hatchlings and juveniles less than 50 mm SVL was white or whitish and faintly banded or boldly banded.

### Morphological analyses

All statistical analyses were performed using the base statistical software in RStudio v. 1.2.1335 (RStudio Team 2018). To remove potential effects of allometry, mensural characters were scaled to SVL using the following allometric equation: X_adj_ = X-β(SVL-SVL_mean_), where X_adj_ = adjusted value; X = measured value; β = unstandardized regression coefficient for each OTU; SVL = measured snout-vent length; SVL_mean_ = overall average SVL of each OTU (Thorpe 1975, 1983; Turan 1999; Lleonart et al. 2000). Male and female measurements were analyzed separately to remove potential effects of sexual dimorphism. For morphological analyses, TL (tail length) was excluded due to their different conditions (e.g., original, broken, and regenerated). Importantly, the following type material and topotypic specimens were included in the analysis: *C. macrotuberculatus* (holotype and three paratypes) and *C. phuketensis* (holotype, paratype and three topotypes). Prior to the morphological analyses, individuals were assigned on the basis of molecular data except *C. phuketensis* based on its distribution into three groups (= species): *C. macrotuberculatus*, *C. phuketensis*, and *C. pulchellus*.

Principal component analysis (PCA) was implemented in the R package FactoMineR (Lê et al. 2008) to discover or reduce dimensionality of the original character variables in order to recover characters bearing the highest degree of variation among groups. Fifteen scaled morphometric and seven meristic characters (scalations) were concatenated and used for the PCA analyses separately by sex. For females, femoroprecloacal pore counts were excluded from the PCA due to their presence in only males.

For univariate analyses, all transformed mensural characters were tested for normality using the Shapiro-Wilk Test. Equality of variances was tested using F-tests. Morphological differences of both males and females between *C. macrotuberculatus* and *C. phuketensis* were examined using a *t*-test (for normally distributed and equal variance data), Welch’s *t*-test (for unequal variance data) and Mann-Whitney U test (for non-normally distributed data) at a significant level of 95%.

## Results

### Phylogenetic relationships

**Figure 2.**
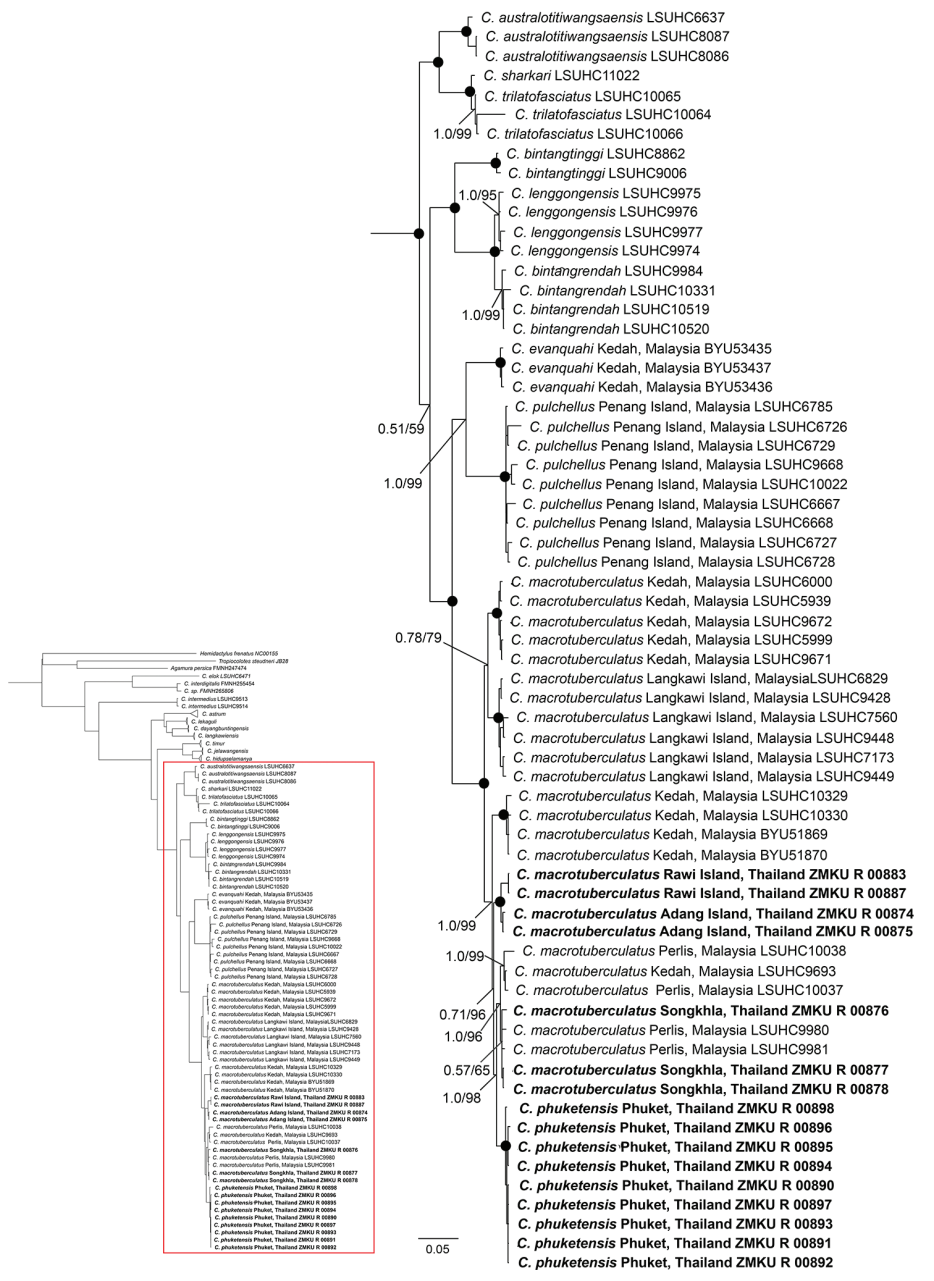
Reconstructed phylogenetic relationships of the *Cyrtodactylus pulchellus* complex based on 1,453 bp of ND2 and flanking tRNAs. The phylogenetic tree is from the Maximum Likelihood analysis with Bayesian posterior probabilities (BPP) and ultrafast bootstrap support values (UFB), respectively. Black circles represent nodes supported by BPP and UFB of 1.0 and 100. Samples in bold are new sequence from this study.

The aligned matrix contained 1,453 bp of ND2 gene and its flanking tRNAs for 101 samples of the *C. pulchellus* complex including outgroups (Table 1). The standard deviation of split frequencies among the two Bayesian runs was 0.003263, and the Estimated Sample Size (ESS) of all parameters were ≥ 200. The BI and ML analyses generated similar topologies and strong nodal support for most clades, and only the ML tree is shown (Fig. 2). According to phylogenetic analyses, *C. phuketensis* is nested within *C. macrotuberculatus* with strong support (1.00 BPP, 100 UFB), thus rendering *C. macrotuberculatus* paraphyletic. *Cyrtodactylus macrotuberculatus* (including *C. phuketensis*) was recovered as sister lineage to a clade containing *C. pulchellus* and *C. evanquahi*. Uncorrected pairwise sequence divergence (*p*-distance) between *C. phuketensis* and this sister lineage was higher than 8.45% and within the *C. phuketensis* and *C. macrotuberculatus* clade, it ranged from 1.45–4.20% (mean 2.63%; Table 2). The *p*-distance within species ranged from 0.00–0.36% (mean 0.14%) for *C. phuketensis* and 0.00–4.38% (mean 2.48%) for *C. macrotuberculatus*.

**Table 2.** Percentage uncorrected pairwise sequence divergence (*p*-distance) for *Cyrtodactylus macrotuberculatus*, *C. phuketensis*, and closely related species calculated from 1,453 base pairs of the mitochondrial gene ND2 and the flanking tRNAs. Numbers in bold represent the mean and the range of within species *p*-distances.

	Species	*N*	1	2	3	4
1	*C. pulchellus*	9	**1.02 (0.14–2.20)**			
2	*C. evanquahi*	3	7.42 (6.64–8.38)	**0.24 (0.14**–**0.36)**		
3	*C. macrotuberculatus*	27	8.93 (7.47–10.48)	8.08 (6.64–8.38)	**2.48 (0.00**–**4.38)**	
4	*C. phuketensis*	9	9.41 (8.71–10.29)	8.79 (8.45–8.95)	2.63 (1.45–4.20)	**0.14 (0.00**–**0.36)**

### Morphology

**Figure 3.**
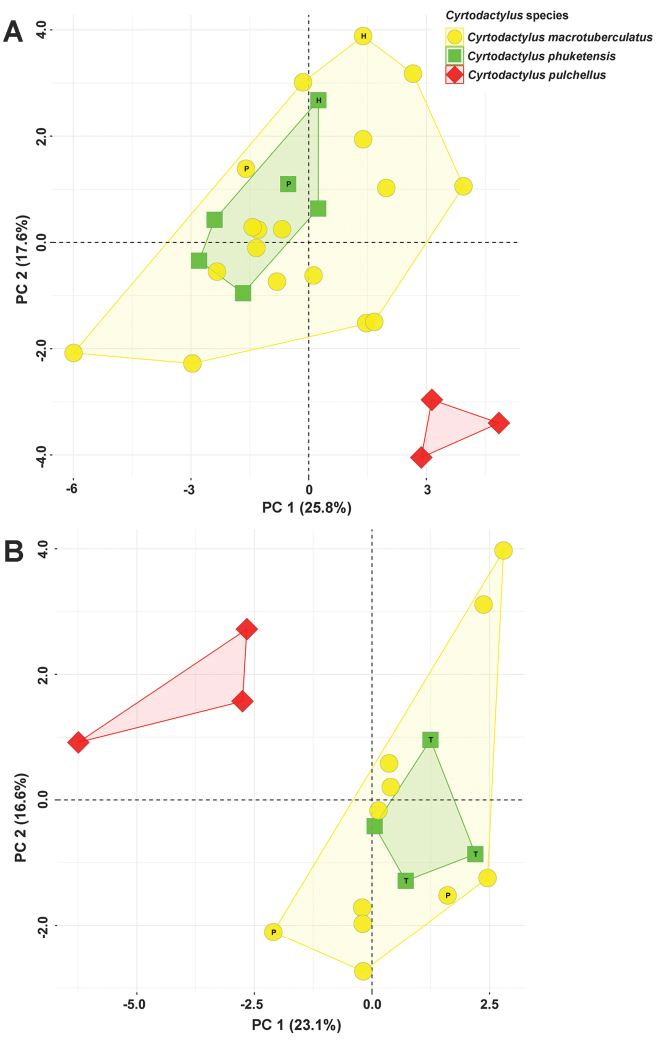
Plots for the first two principal components of morphological characters from **A** males, and **B** females resulting from the principal component analyses of *Cyrtodactylus macrotuberculatus* (yellow circles), *C. phuketensis* (green squares) and *C. pulchellus* (red diamonds). The letters in the scatter plots refer to holotype (= H), paratype (= P) and topotype (= T).

A total of 45 preserved specimens from three species groups (*C. macrotuberculatus* = 29, *C. phuketensis* = 10, and *C. pulchellus* = 6) were used for principal component analysis (Table 3). The PCA of males showed complete overlap between *C. macrotuberculatus* and *C. phuketensis*, and they were completely separated from *C. pulchellus* along the first two principal components (Fig. 3A). The first three principal components of males accounted for 53.17% of the variation. The first principal component (PC1) accounted for 25.78% of the variation and was most heavily loaded on HL_adj_, ES_adj_, EN_adj_ and ventral scales; the PC2 accounted for 17.56% of the variation and was most heavily loaded on TBL_adj_, IO_adj_, supralabial and infralabial scales; and the PC3 accounted for 9.83% of the variation and was loaded most heavily on longitudinal tubercles (Table 3).

**Table 3.** Summary statistics and factor loadings of the principal component analysis from morphological characters for males and females *Cyrtodactylus macrotuberculatus*, *C. phuketensis*, and *C. pulchellus*. Morphological character abbreviations are defined in the Materials and methods. / = data unavailable.

Characters	Males			Females		
	PC1	PC2	PC3	PC1	PC2	PC3
SVL_adj_	0.067	-0.193	-0.291	0.080	-0.043	-0.047
TW_adj_	0.558	0.589	-0.029	-0.823	0.036	0.055
FL_adj_	0.586	-0.425	0.023	-0.040	0.507	-0.474
TBL_adj_	0.467	-0.690	0.027	-0.055	0.452	-0.752
AG_adj_	-0.185	-0.442	0.415	0.685	-0.284	-0.283
HL_adj_	0.784	0.141	-0.033	0.384	0.730	0.436
HW_adj_	0.439	0.569	-0.144	0.232	0.405	-0.163
HD_adj_	0.577	0.539	-0.221	0.516	-0.070	0.040
ED_adj_	0.585	-0.173	0.374	-0.364	0.777	0.337
EE_adj_	0.484	0.314	-0.580	0.581	0.338	-0.056
ES_adj_	0.858	-0.053	-0.017	0.669	0.260	0.455
EN_adj_	0.774	-0.289	0.004	0.726	-0.087	0.133
IO_adj_	0.147	0.662	0.003	0.602	0.117	0.174
EL_adj_	0.351	-0.097	-0.360	0.141	0.190	0.714
IN_adj_	0.527	0.224	-0.184	-0.216	0.150	-0.473
Supralabials	-0.217	0.622	0.270	-0.070	-0.745	0.307
Infralabials	0.218	0.627	0.402	-0.258	-0.690	0.340
Paravertebral tubercles	-0.157	0.472	0.409	-0.287	-0.196	-0.168
Longitudinal tubercles	0.363	0.215	0.642	-0.133	0.215	0.464
Ventral scales	0.761	-0.297	0.338	-0.665	0.497	0.230
4^th^ toe lamellae	0.439	-0.324	-0.217	-0.864	0.095	0.283
Femoroprecloacal pores	0.512	-0.196	0.468	/	/	/
Eigenvalue	5.671	3.864	2.164	4.859	3.484	2.784
Percentage of variance	25.776	17.562	9.834	23.136	16.592	13.258
Cumulative proportion	25.776	43.338	53.172	23.136	39.728	52.986

Along the first two PC plots, the PCA of females revealed complete overlap between *C. macrotuberculatus* and *C. phuketensis*, which were distinctly separated from *C. pulchellus* (Fig. 3B). The first three principal components of females accounted for 52.99% of the variation. The first principal component (PC1) accounted for 23.14% of the variation and was most heavily loaded on TW_adj_, AG_adj_, ES_adj_, EN_adj_, ventral scales and number of the 4^th^ toe lamellae; the PC2 accounted for 16.59% of the variation and was most heavily loaded on HL_adj_, ED_adj_, supralabial and infralabial scales; the PC3 accounted for 13.26% of the variation and was loaded most heavily on TBL_adj_ and EL_adj_ (Table 3).

Summary univariate statistics of morphological characters of adult males and females are shown in Table 4. In adult males, *C. macrotuberculatus* (*N* = 18) and *C. phuketensis* (*N* = 6) were not significantly different in most morphological characters (*t*-tests and Mann-Whitney *U* tests, *p* = 0.1765–0.9523) except only IO_adj_ (*t*-test, *p* = 0.0256). In adult females, *C. macrotuberculatus* (*N* = 11) and *C. phuketensis* (*N* = 4) were not significantly different in twelve morphological characters (*t*-tests and Welch’s *t*-test, *p* = 0.2325–0.9626) whereas only three characters were significantly different which are TBL_adj_ (*t*-test, *p* = 0.0495), AG_adj_ (Mann-Whitney U tests, *p* = 0.0176) and IN_adj_ (Welch’s *t*-test, *p* = 0.0129).

**Table 4.** Comparisons of fifteen morphological characters between *Cyrtodactylus macrotuberculatus* and *C. phuketensis*. Data are given as mean and standard deviation, followed by range in parentheses. Morphological character abbreviations are defined in the Materials and methods. Key: ^a^ tested by Welch’s *t*-test, ^b^ tested by Mann-Whitney U test, * significance level at *p* < 0.05.

Characters	Males				Females			
	*C. macrotuberculatus*	*C. phuketensis*	*t*-tes*t*	*p*	*C. macrotuberculatus*	*C. phuketensis*	*t*-tes*t*	*p*
	*N* = 18	*N = 6*			*N* = 11	*N = 4*		
SVL	105.8 ± 8.9	105.3 ± 10.0	0.112	0.9122	103.7 ± 10.1	104.4 ± 16.0	-0.098	0.9237
	(88.9–117.9)	(93.2–115.3)			(84.1–115.7)	(84.8–117.6)		
TW	9.5 ± 0.9	9.6 ± 1.2	-0.061	0.9523	8.1 ± 1.4	8.2 ± 2.1	-0.352	0.7307
	(8.0–11.6)	(7.9–10.9)			(5.9–10.3)	(6.2–10.5)		
FL	17.2 ± 1.5	17.2 ± 2.0	-0.098	0.9229	16.8 ± 1.6	16.8 ± 2.7	0.048	0.9626
	(14.2–18.9)	(14.4–19.0)			(13.6–18.3)	(13.3–19.3)		
TBL	20.3 ± 1.6	20.6 ± 2.6	-0.631	0.5348	19.9 ± 1.8	20.4 ± 3.2	-2.166	0.0495*
	(17.3–22.8)	(17.4–19.0)			(16.3–21.9)	(16.5–23.2)		
AG	50.4 ± 4.6	51.5 ± 5.9	-1.034	0.3125	50.7 ± 4.5	53.4 ± 7.9	4^b^	0.0176*
	(41.3–58.6)	(44.7–57.4)			(42.0–56.0)	(43.4–60.2)		
HL	29.4 ± 2.2	29.2 ± 2.7	0.590	0.5613	28.5 ± 3.1	28.4 ± 4.4	0.575^a^	0.5758
	(24.6–33.3)	(25.6–31.7)			(22.8–32.3)	(23.1–32.1)		
HW	20.0 ± 1.8	19.8 ± 2.7	0.416	0.6816	19.0 ± 1.9	18.7 ± 2.6	0.815	0.4297
	(16.7–22.9)	(16.3–22.5)			(15.6–21.0)	(15.8–21.4)		
HD	12.0 ± 1.3	12.2 ± 1.9	-0.840	0.4100	11.3 ± 1.3	11.5 ± 2.0	-1.054	0.3109
	(9.7–14.1)	(9.9–14.2)			(9.0–13.4)	(9.1–13.5)		
ED	6.9 ± 0.6	6.7 ± 0.5	1.397	0.1765	6.7 ± 0.7	6.5 ± 1.1	1.253	0.2325
	(5.8–7.9)	(5.6–7.0)			(5.6–7.6)	(7.4–9.6)		
EE	8.6 ± 1.0	8.6 ± 1.0	62^b^	0.6261	8.5 ± 0.8	8.6 ± 1.1	-0.106	0.9171
	(6.5–9.8)	(7.1–9.4)			(7.0–9.4)	(7.4–9.6)		
ES	11.7 ± 1.0	11.7 ± 1.1	56^b^	0.9225	11.5 ± 1.2	11.4 ± 1.8	0.271^a^	0.7912
	(10.0–13.6)	(10.3–12.9)			(9.3–13.1)	(9.2–12.9)		
EN	8.7 ± 0.7	8.7 ± 0.7	0.090	0.9288	8.6 ± 0.9	8.5 ± 1.3	0.610	0.5521
	(7.3–9.8)	(7.6–9.5)			(6.8–9.8)	(6.8–9.6)		
IO	5.1 ± 0.6	4.8 ± 0.6	2.394	0.0256*	4.8 ± 0.8	4.6 ± 0.9	0.835	0.4186
	(4.0–6.3)	(4.1–5.5)			(3.4–5.7)	(3.6–5.5)		
EL	2.3 ± 0.3	2.2 ± 0.4	0.504	0.6192	2.4 ± 0.4	2.2 ± 0.5	1.125	0.281
	(1.7–2.8)	(1.4–2.6)			(1.6–3.0)	(1.5–2.5)		
IN	2.2 ± 0.3	2.3 ± 0.3	-0.352	0.7282	2.0 ± 0.5	2.3 ± 0.3	-1.785^a^	0.0129*
	(1.7–2.7)	(1.7–2.5)			(1.3–2.9)	(1.9–2.6)		

Sumontha et al. (2012) distinguished *C. phuketensis* from *C. macrotuberculatus* by using the number of subdigital lamellae on the fourth toe, number of dark body bands, and the presence of a precloacal groove in females. Based on examination of the type material and newly collected specimens of *C. phuketensis* from Phuket Island, these diagnostic characters overlap with those characters of *C. macrotuberculatus*. In this study, *C. phuketensis* has 19–21 total subdigital lamellae on the fourth toe (*vs.* 19–23 in *C. macrotuberculatus*); three or four dark body bands (*vs.* three or four in *C. macrotuberculatus*), and no precloacal groove in females (also absent in *C. macrotuberculatus*; Table 5).

**Table 5.** Summarized diagnostic characters of *Cyrtodactylus macrotuberculatus* and *C. phuketensis* taken from original descriptions (Grismer and Ahmad 2008; Sumontha et al. 2012) and this study based on type materials and newly additional specimens. / = data unavailable.

	*C. macrotuberculatus*	*C. phuketensis*	*C. macrotuberculatus*	*C. phuketensis*
	(Grismer and Ahmad, 2008)	(Sumontha et al., 2012)	This study	This study
Supralabials	10–12	12–13	9–12	9–13
Infralabials	8–11	9–10	7–11	7–10
Tuberculation	Prominent	Prominent	Prominent	Prominent
Tubercles on ventral surface of forelimbs	Yes	Yes	Yes	Yes
Tubercles in gular region	Yes	Yes	Yes	Yes
Ventrolateral fold tuberculate	Yes	Yes	Yes	Yes
Paravertebral tubercles	40–47	40–43	34–49	39–45
Longitudinal rows of tubercles	22–26	23–24	19–27	20–24
Ventral scales	19–22	22–24	17–28	20–24
4^th^ toe lamellae	21–23	19	19–23	19–21
Femoroprecloacal pores	35–37	33–36	28–42	31–33
Precloacal groove present in females	No	Yes	No	No
Precloacal depression in males	No	No	Deep	Deep
No. of body bands	4	3 (3.5 one individual)	3–4	3–4 (3+1 incomplete band)
Body band/interspace ratio	/	/	0.95–1.74	1.02–1.50
Dorsum bearing scattered pattern of white tubercles	No	No	No	No
Hatchlings/juveniles with white tail tip	No	No	No	No
Dark caudal bands on original tail	/	8	7–10	7–8
White caudal bands in adults immaculate	/	/	No	No
Maximum SVL	120.0	114.7	117.87	117.61
Sample size	5	3	29	10

### Systematics

The phylogenetic analyses recovered *C. phuketensis* as being nested within the *C. macrotuberculatus* and bearing a low genetic divergence (mean 2.63%) which was similar to that within *C. macrotuberculatus* populations (mean 2.48%). In concordance, the statistical analyses of meristic and mensural characters of *C. phuketensis* widely overlap with those of *C. macrotuberculatus*. Based on these data, we propose that *C. phuketensis* from Phuket Island, Phuket Province is a junior synonym of *C. macrotuberculatus* which can be recognized as follows.

### Taxonomy

#### Cyrtodactylus macrotuberculatus

Taxon classificationAnimaliaSquamataGekkonidae

Grismer and Ahmad, 2008

D765E47B-9F30-541B-8C53-5E2EC6C7D3F3

Figure 4

Cyrtodactylus macrotuberculatus Grismer & Ahmad, 2008: 55; Grismer 2011: 406; Grismer et al. 2012: 45.
Cyrtodactylus phuketensis Sumontha et al., 2012: 62.

##### Type specimens.

***Holotype*** (adult male, ZRC 2.6754) from Malaysia, Kedah, Pulau Langkawi, Gunung Raya; ***Paratypes***: Malaysia, Kedah, Pulau Langkawi, Gunung Raya: ZRC 2.6755–2.6756, Telaga Tujuh: ZRC 2.6757, Lubuk Semilang: ZRC 2.6758.

##### Additional specimens examined

**(including types of *C. phuketensis*). Malaysia** – Kedah, Pulau Langkawi, Gunung Raya: LSUHC 09428–09429, LSUHC 09432; Perlis, Perlis State Park: LSUHC 09981, LSUHC 10097, ZRC 2.4869. **Thailand** – Satun Province, Mueang Satun District, Adang Island: ZMKU R 00871–00875, ZMKU R 00879–00882, Rawi Island: ZMKU R 00883–00889; Songkhla Province, Hat Yai District, Chalung Sub-district: ZMKU R 00876–00878; Phuket Province, Thalang District: PSUZC-RT 2010.58, THNHM 15378, ZMKU R 00894–00896, Kathu District: ZMKU R 00890–00893, ZMKU R 00897–00898 (Table 1).

##### Expanded diagnosis.

*Cyrtodactylus macrotuberculatus* can be separated from all other species of *C. pulchellus* complex by having the following combination of characters (Table 6): (1) maximum SVL 117.9 mm (mean 105.0 ± SD 9.8, *N* = 39); (2) 9–13 supralabial and 7–11 infralabial scales; (3) prominent tuberculation on body; (4) tubercles on ventral surface of forelimbs, gular region, in ventrolateral body folds, and anterior one-third of tail; (5) 34–49 paravertebral tubercles; (6) 19–27 longitudinal tubercle rows; (7) 17–28 ventral scales; (8) 19–23 subdigital lamellae on the fourth toe; (9) 28–42 femoroprecloacal pores in males; (10) deep precloacal groove in males; (11) three or four dark dorsal body bands; (12) body band wider than interspace; (13) 7–10 (*N* = 12) ringed dark caudal bands on original tail; (14) white caudal bands infused with dark pigmentation in adults; (15) posterior portion of tail in hatchlings and juveniles bands not white.

**Table 6.** Morphological measurement (mm), meristic and non-meristic data from males and females of *Cyrtodactylus macrotuberculatus*. Morphological character abbreviations are defined in the Materials and methods.

Characters	Adult males (*N* = 24)		Adult females (*N* = 15)		All (*N* = 39)	
	Mean ± SD	(Min–Max)	Mean ± SD	(Min–Max)	Mean ± SD	(Min–Max)
SVL	105.7 ± 9.0	(88.9–117.9)	103.9 ± 11.3	(84.1–117.6)	105.0 ± 9.8	(84.1–117.9)
TW	9.5 ± 1.0	(7.9–11.6)	8.2 ± 1.5	(5.9–10.5)	9.0 ± 1.4	(5.9–11.6)
FL	17.2 ± 1.6	(14.2–19.0)	16.8 ± 1.8	(13.3–19.3)	17.1 ± 1.7	(13.3–19.3)
TBL	20.4 ± 1.9	(17.3–23.5)	20.0 ± 2.1	(16.3–19.3)	20.2 ± 2.0	(16.3–23.5)
AG	50.7 ± 4.9	(41.3–58.6)	51.4 ± 5.4	(42.0–60.2)	51.0 ± 5.0	(41.3–60.2)
HL	29.4 ± 2.3	(24.6–33.3)	28.5 ± 3.3	(22.8–32.3)	29.0 ± 2.7	(22.8–33.3)
HW	20.0 ± 2.0	(16.3–22.9)	18.9 ± 2.0	(15.6–21.4)	19.6 ± 2.0	(15.6–22.9)
HD	12.1 ± 1.4	(9.7–14.2)	11.3 ± 1.4	(9.0–13.5)	11.8 ± 1.4	(9.0–14.2)
ED	6.8 ± 0.6	(5.6–7.9)	6.7 ± 0.8	(5.2–7.5)	6.8 ± 0.7	(5.2–7.9)
EE	8.6 ± 1.0	(6.5–9.8)	8.5 ± 0.9	(7.0–9.6)	8.6 ± 0.7	(5.2–7.9)
ES	11.7 ± 1.0	(10.0–13.6)	11.5 ± 1.3	(9.2–13.1)	11.6 ± 1.1	(9.2–13.6)
EN	8.7 ± 0.7	(7.3–9.8)	8.6 ± 1.0	(6.8–9.8)	8.6 ± 0.8	(6.8–9.8)
IO	5.0 ± 0.6	(4.0–6.3)	4.8 ± 0.8	(3.4–5.7)	4.8 ± 0.7	(3.4–6.3)
EL	2.3 ± 0.3	(1.4–2.8)	2.3 ± 0.4	(1.5–3.0)	2.3 ± 0.4	(1.4–3.0)
IN	2.2 ± 0.5	(1.7–2.7)	2.1 ± 0.5	(1.3–2.9)	2.2 ± 0.4	(1.3–2.9)
HL/SVL	0.28 ± 0.01	(0.27–0.30)	0.27 ± 0.01	(0.26–0.29)	0.28 ± 0.01	(0.26–0.30)
HW/HL	0.68 ± 0.03	(0.62–0.74)	0.67 ± 0.02	(0.62–0.70)	0.67 ± 0.03	(0.62–0.74)
HD/HL	0.41 ± 0.02	(0.37–0.45)	0.40 ± 0.01	(0.38–0.42)	0.41 ± 0.02	(0.37–0.45)
ES/HL	0.40 ± 0.01	(0.37–0.41)	0.40 ± 0.00	(0.39–0.41)	0.40 ± 0.01	(0.37–0.41)
ED/HL	0.23 ± 0.01	(0.21–0.27)	0.23 ± 0.01	(0.22–0.25)	0.23 ± 0.01	(0.21–0.27)
EL/HL	0.08 ± 0.01	(0.05–0.10)	0.08 ± 0.01	(0.05–0.10)	0.08 ± 0.01	(0.05–0.10)
AG/SVL	0.48 ± 0.02	(0.43–0.51)	0.50 ± 0.01	(0.47–0.52)	0.49 ± 0.02	(0.43–0.52)
FL/SVL	0.16 ± 0.00	(0.15–0.17)	0.16 ± 0.00	(0.16–0.17)	0.16 ± 0.00	(0.15–0.17)
TBL/SVL	0.19 ± 0.01	(0.18–0.21)	0.19 ± 0.00	(0.18–0.20)	0.19 ± 0.01	(0.18–0.21)
TL/SVL	1.29 ± 0.04	(1.23–1.35)	1.27 ± 0.04	(1.24–1.34)	1.28 ± 0.04	(1.23–1.35)
Supralabials	9–13		9–12		9–13	
Infralabials	7–11		7–11		7–11	
Tuberculation	Prominent		Prominent		Prominent	
Tubercles on ventral surface of forelimbs	Yes		Yes		Yes	
Tubercles in gular region	Yes		Yes		Yes	
Ventrolateral fold tuberculate	Yes		Yes		Yes	
Paravertebral tubercles	37–49		34–47		34–49	
Longitudinal rows of tubercles	19–27		19–26		19–27	
Ventral scales	17–28		19–26		17–28	
4^th^ toe lamellae	19–23		19–23		19–23	
Femoroprecloacal pores	28–42		No		28–42	
Precloacal depression	Yes		No		Only in males	
No. of body bands	3 or 4		3 or 4		3 or 4	
Body band/interspace ratio	0.95–1.75		1.03–1.62		0.95–1.74	
Dark caudal bands on original tail	7–9		7–10		7–10	

##### Description of adult males.

SVL of adult males range from 88.9–117.9 mm (mean 105.7, *N* = 24); head moderate in length (HL/SVL 0.27–0.30), width (HW/HL 0.62–0.74), somewhat flattened (HD/HL 0.37–0.45), distinct from neck, triangular in dorsal profile; lores concave; frontal and prefrontal regions deeply concave; canthus rostralis sharply rounded; snout elongate (ES/HL 0.37–0.41), rounded in dorsal profile, laterally constricted; eye large (ED/HL 0.21–0.27); ear opening elliptical, moderate in size (EL/HL 0.05–0.10) obliquely oriented; eye to ear distance greater than diameter of eye; rostral rectangular, divided dorsally by an inverted Y or I-shaped furrow, bordered posteriorly by large left and right supranasals and small internasal, bordered laterally by external nares and first supralabials; external nares bordered anteriorly by rostral, dorsally by one large anterior supranasal, posteriorly by two postnasals, ventrally by first supralabial; 9–13 rectangular supralabials extending to just beyond upturn of labial margin, tapering abruptly below midpoint of eye; 7–11 infralabials not tapering in size posteriorly; scales of rostrum and lores slightly raised, larger than granular scales on top of head and occiput, those on posterior portion of canthus rostralis slightly larger; scales on top of head and occiput intermixed with enlarged tubercles; large, boney frontal ridges bordering orbit confluent with boney, transverse, parietal ridge; dorsal superciliaries elongate, smooth, largest anteriorly; mental triangular, bordered laterally by first infralabials and posteriorly by left and right trapezoidal postmentals that contact medially for 40–50% of their length posterior to mental; single row of slightly enlarged, elongate sublabials extending posteriorly to 5^th^–7^th^ infralabial; small, granular, gular scales intermixed with numerous large, conical tubercles grading posteriorly into larger, conical tubercles on throat which abruptly transition into large, flat, smooth, imbricate, pectoral and ventral scales.

Body relatively short (AG/SVL 0.43–0.51) with well-defined, tuberculate, ventrolateral folds; dorsal scales small, granular, interspersed with large, trihedral, regularly arranged, keeled tubercles separated by no more than three granules at their base; tubercles extend from top of head onto approximately one-half of tail but not onto regenerated tail; tubercles on occiput and nape relatively small, those on body largest; approximately 19–27 longitudinal rows of dorsal tubercles at the mid body; approximately 37–49 paravertebral tubercles; 17–28 flat, imbricate, ventral scales and much larger than dorsal scales; precloacal scales large, smooth; deep precloacal groove (= depression).

Forelimbs moderate in stature, relatively short (FL/SVL 0.15–0.17); virtually no granular scales on dorsal surface of forelimbs, only large, trihedral, keeled tubercles; palmar scales slightly rounded; digits well-developed, inflected at basal, interphalangeal joints; subdigital lamellae nearly square proximal to joint inflection, only slightly expanded distal to inflection; digits more narrow distal to joints; claws well-developed, sheathed by dorsal and ventral scale; hind limbs more robust than forelimbs, moderate in length (TBL/SVL 0.18–0.21), virtually no granular scales on dorsal surfaces of hind limbs, only large, trihedral, keeled tubercles; ventral scales of thigh flat, smooth, imbricate; ventral, tibial scales flat, imbricate, slightly keeled; two rows of enlarged, flat, imbricate, femoroprecloacal scales extend from knee to knee through precloacal region where they are continuous with enlarged, pore-bearing precloacal scales; 28–42 contiguous, pore-bearing femoroprecloacal scales forming an inverted T bearing a deep, precloacal groove; eight to eleven pores bordering groove; postfemoral scales immediately posterior to the pore-bearing scale row conical, forming an abrupt union on posteroventral margin of thigh; plantar scales low, slightly rounded; digits well-developed, inflected at basal, interphalangeal joints; subdigital lamellae proximal to joint inflection nearly square, only slightly expanded distal to inflection; digits more narrow distal to joints; claws well-developed, sheathed by a dorsal and ventral scale; 19–23 subdigital lamellae on the 4^th^ toe.

Original tail (TL/SVL) moderate in proportions, 123–135% of SVL (mean 128, *N* = 12), 7.9–11.6 mm in width at base, tapering to a point; dorsal scales at base of tail square, smooth, flat, subimbricate, lacking tubercle on regenerated tail; median row of transversely enlarged, subcaudal scales; shallow caudal furrow; two to five small, postcloacal tubercles at base of tail on hemipenial swellings; all postcloacal scales flat, large, imbricate.

**Figure 4.**
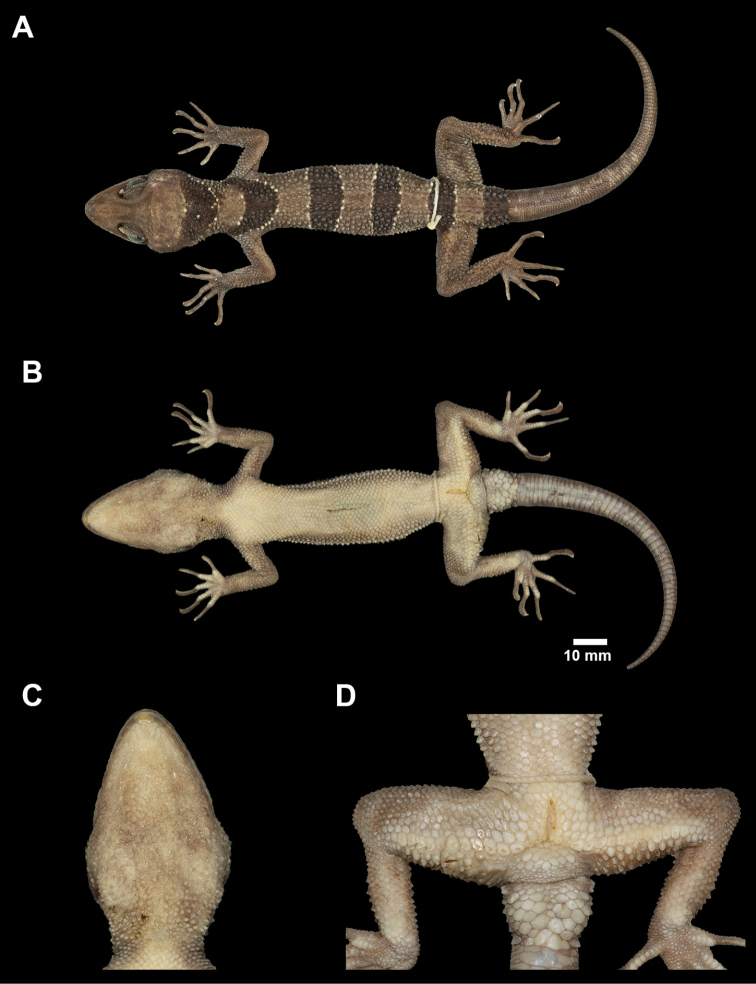
Male holotype of *Cyrtodactylus macrotuberculatus* from Pulau Langkawi, Kedah, Peninsular Malaysia (ZRC 2.6754) in preservative **A** dorsal view **B** ventral view **C** tuberculate gular region and throat, and **D** enlarge femoroprecloacal scale row and pores.

##### Coloration of adult male ZMKU R 00871 in life

**Figure 5.**
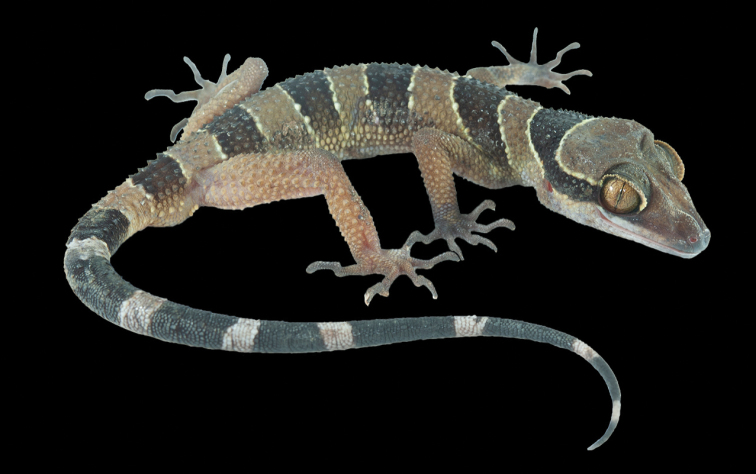
Adult male *Cyrtodactylus macrotuberculatus* from Adang Island, Satun Province, Thailand (ZMKU R 00871) in life.

**(Fig. 5)**. Ground color of head, body, limbs, and dorsum light-brown to yellowish brown; wide, dark-brown nuchal band edged anteriorly and posteriorly by thin, creamy-white lines bearing tubercles extends from posterior margin of one eye to posterior margin of other eye; four similar body bands between nuchal loop and hind limb insertions edged anteriorly and posteriorly by thin, creamy-white lines bearing tubercles, first band terminates at shoulders, second and third bands terminate just dorsal to ventrolateral folds, the fourth band terminates at femurs; dark body bands slightly larger than light-colored interspaces; one additional dark-brown band posterior to hind limbs; original portion of tail bearing eight ringed, dark-colored bands separated by seven, narrower, off-white bands infused with dark pigmentation; ventral surfaces of head smudged with brown; abdomen and limbs beige, slightly darker, lateral regions.

##### Coloration in preservative

**Figure 6.**
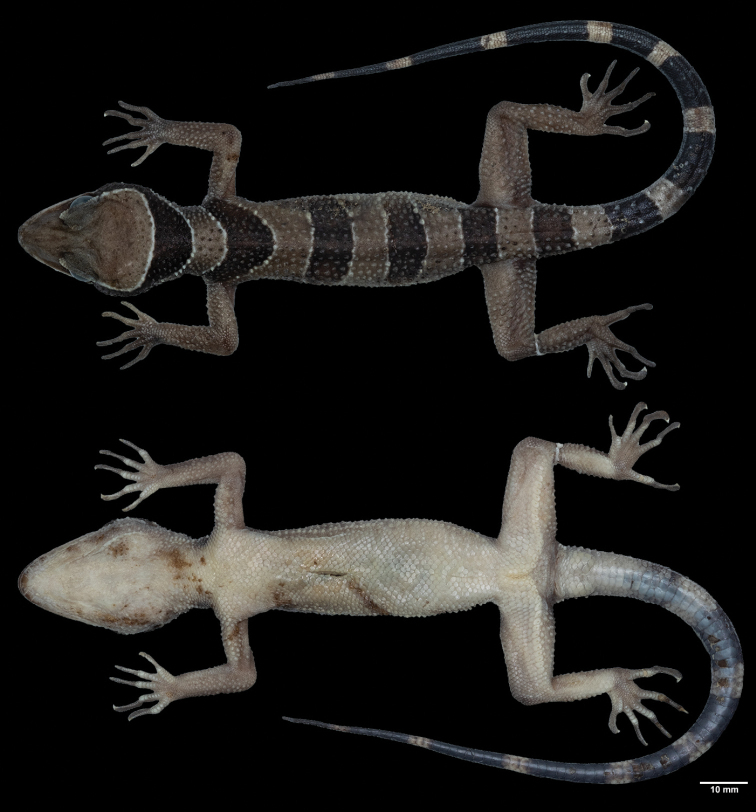
Adult male *Cyrtodactylus macrotuberculatus* from Adang Island, Satun Province, Thailand (ZMKU R 00871) in preservative **A** dorsal and **B** ventral views.

**(Fig. 6).** Color pattern of head, body, limbs, and tail similar to that in life with some fading. Ground color of head, body, limbs, and dorsum tan; dark body and dark caudal bands lighter than in life.

##### Variation.

**Figure 7.**
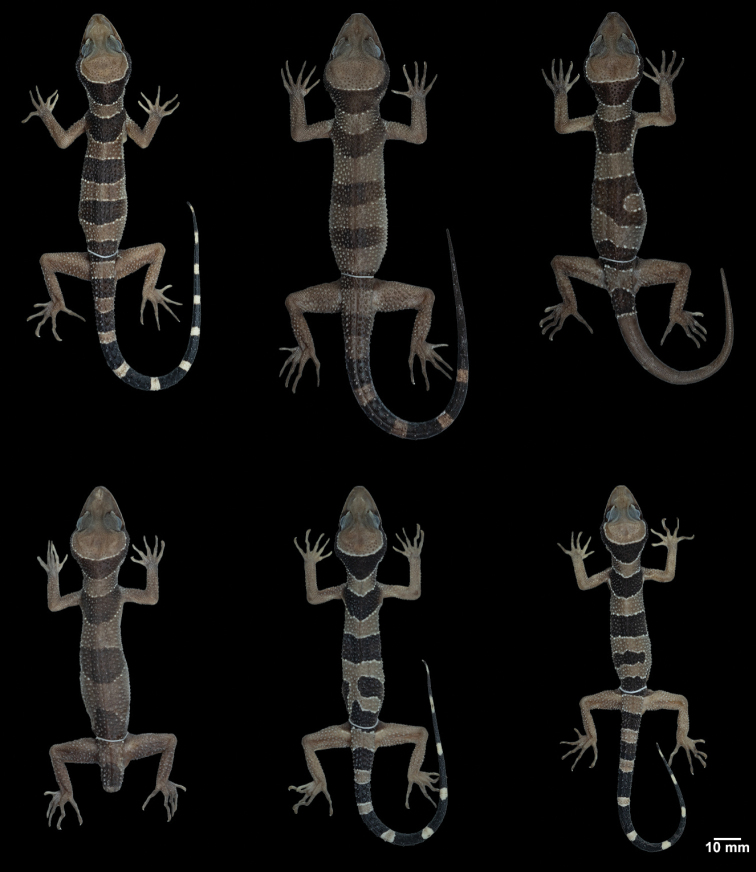
Variation in dorsal body band pattern of *Cyrtodactylus macrotuberculatus* from Thailand. From left to right, upper: ZMKU R 00878, ZMKU R 00873 from Adang Island, Satun Province; and ZMKU R 00887 from Rawi Island, Satun Province. Lower: ZMKU R 00889 from Rawi Island, Satun Province; ZMKU R 00896 and ZMKU R 00895 from Phuket Province.

**Figure 8.**
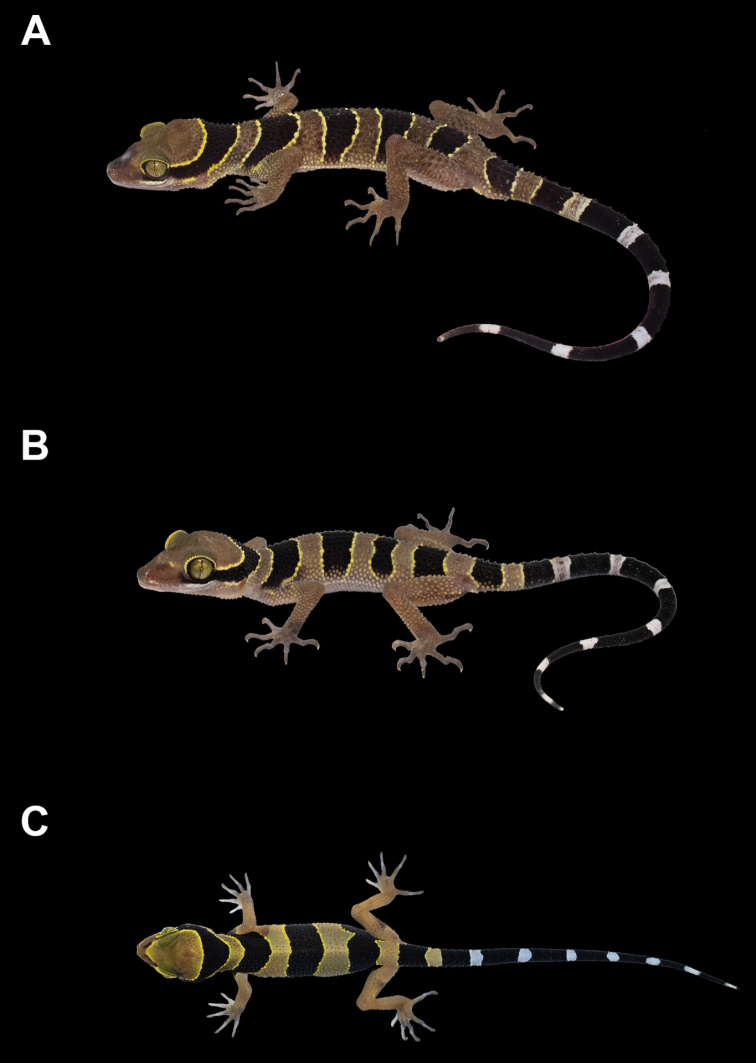
Color in life of *Cyrtodactylus macrotuberculatus* from Thailand **A** adult male ZMKU R 00883 from Rawi Island, Satun Province **B** subadult female ZMKU R 00895 from Thalang District, Phuket Province, and **C** juvenile ZMKU R 00898 from Kathu District, Phuket Province.

**Figure 9.**
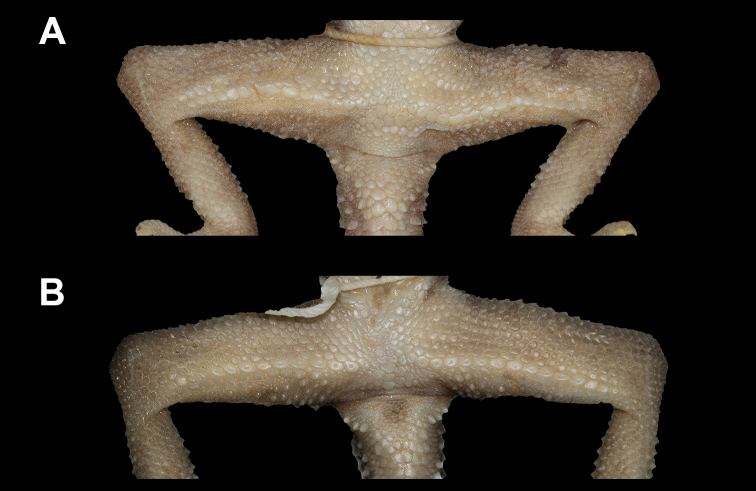
Precloacal region in female specimens of *Cyrtodactylus macrotuberculatus***A** paratype ZRC 2.6758, from Telaga Tujuh, Pulau Langkawi, Malaysia, and **B**ZMKU R 00896 from Thalang District, Phuket Province, Thailand.

*Cyrtodactylus macrotuberculatus* usually varies in coloration and banding pattern (Figs 7–8). In females, a precloacal groove and pores are absent (Fig. 9). PSUZC-RT 2010.58 and THNHM 15378 have a shallow precloacal groove. Three dark body bands occur in PSU 2010.58, THNHM 15378, ZMKU R 00889–00894 and ZMKU R 00897. In ZMKU R 00887, the second dorsal band bifurcates just dorsal to the ventrolateral fold. ZMKU R 00895 has four bands and the third band is incomplete. The third body band in ZMKU R 00896 is broken on the left of the midline and contacts the fourth body band bilaterally. Nuchal loop and body bands of ZMKU R 00883, ZMKU R 00895, and ZMKU R 00898 edged anteriorly and posteriorly by thin, light-yellow lines and tubercles; and dorsal superciliaries are light-yellow (Fig. 8). Variation in morphometric and meristic data are shown in Table 6.

##### Distribution.

*Cyrtodactylus macrotuberculatus* is distributed on the mainland and only known from one island in Peninsular Malaysia and southern Thailand (Fig. 1). This species is known from Pulau Langkawi (Gunung Raya, Telaga Tujuh, Gunung Machinchang, and Lubuk Semilang), Kedah, Peninsular Malaysia (Grismer and Ahmad 2008). Other populations are found from Peninsular Malaysia; Kedah (Bukit Wang, Gunung Jerai, Hutan Lipur Sungai Tupah, Kuala Nerang, and Ulu Muda) and Perlis (Bukit Chabang, Chuping and Perlis State Park; [Grismer 2011; Grismer et al. 2012; Quah et al. 2019]). In Thailand, *C. macrotuberculatus* was recorded from Phatthalung Province (Grismer et al. 2012); Phuket Province, Kathu District (Kathu Waterfall) and Thalang District (Thep Krasatti Sub-district, previously type locality of *C. phuketensis*); Satun Province, La-ngu District, and Mueang Satun District (Adang and Rawi Islands); Songkhla Province, Rattaphum District (Grismer et al. 2012) and Hat Yai District (Ton Nga Chang Waterfall).

##### Natural history.

**Figure 10.**
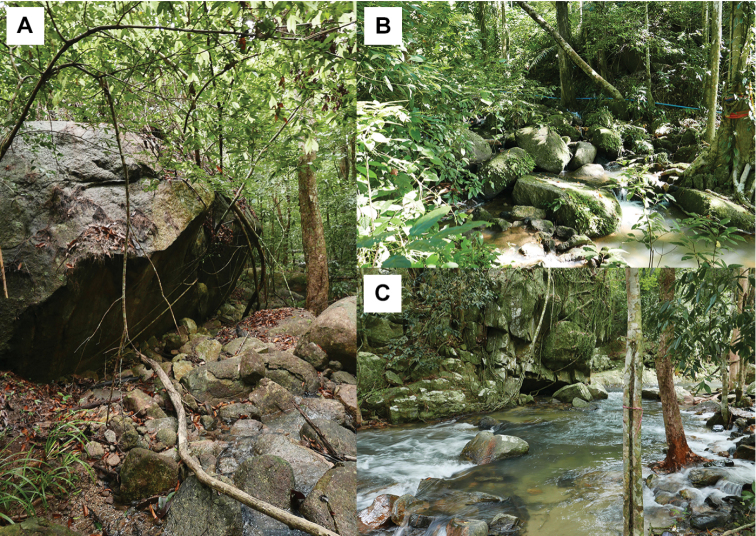
Habitats of *Cyrtodactylus macrotuberculatus* in Thailand **A** Adang Island, Satun Province **B** Kathu District, Phuket Province, and **C** Hat Yai District, Songkhla Province.

Based on specimens in Thailand, all individuals were found in similar habitat type, lowland forest habitat along granitic rock streams and surrounding areas (elevation 7–186 m asl) during a night survey (1900–2200; Fig. 10). The geckos were found mostly on rock boulders, vegetation (trunk of tree, buttress root, rotting wood and vines), and sometimes on the ground with leaf litter and high humidity (26.3–30.8 °C in temperature, 73.8–100% in relative humidity). Gravid female (ZMKU R 00876) contained four eggs during December. One juvenile (ZMKU R 00898, 56.50 mm in SVL) was found on a tree trunk in January. The varied microhabitats within which this species occurs, are consistent with its characterization as a habitat generalist (Grismer et al. 2020, 2021b) and may account for its wide peninsular and insular distribution relative to other species of the *pulchellus* group whose distributions are much less extensive or site-specific (Grismer et al. 2012, 2014, 2016; Quah et al. 2019; Wood et al. 2020).

In Thailand, *C. macrotuberculatus* were found sympatric with other gecko species, *Cnemaspis adangrawi*Ampai et al., 2019 on Adang and Rawi Islands, Satun Province (Ampai et al. 2019); *Cnemaspis phuketensis* Das and Leong, 2004, *Cyrtodactylus oldhami* Theobald, 1876, and Gekko (Ptychozoon) tokehos Grismer et al., 2019 at Kathu and Thalang District, Phuket Province; G. (P.) tokehos, *Cnemaspis kumpoli* Taylor, 1963, and *Gehyra mutilata* (Weigmann, 1834) at Hat Yai District, Songkhla Province.

##### Comparison.

*Cyrtodactylus macrotuberculatus* is distinguished from all other 15 species in the *C. pulchellus* complex by a combination of morphological characters (Table 7). It differs from all other species by having prominent tuberculation on the body; tubercles on ventral surface of forelimbs, gular region, and in ventrolateral body folds; 34–49 paravertebral tubercles; 19–27 longitudinal tubercle rows; 17–28 ventral scales; 19–23 subdigital lamellae on the fourth toe; 28–42 femorprecloacal pores in males; deep precloacal groove in males; no scattered white spots on dorsum; 7–10 dark-ringed caudal bands on original tail; white caudal bands on original tail infused with dark pigmentation in adults. Additional comparisons between *C. macrotuberculatus* and other species in *C. pulchellus* complex are in Table 7.

**Table 7.** Diagnostic characters of *Cyrtodactylus macrotuberculatus* and related species within *C. pulchellus* complex. W = weak; P = prominent; / = data unavailable. Some information was collected from the following literature (Grismer et al. 2012, 2014, 2016; Quah et al. 2019; Wood et al. 2020).

	* australotitiwangsaensis *	* bintangtinggi *	* bintangrendah *	* evanquahi *	* lenggongensis *	* pulchellus *	* sharkari *	* trilatofasciatus *	*macrotuberculatus* (this study)	* astrum *	* dayangbuntingensis *	* hidupselamanya *	* langkawiensis *	* lekaguli *	* jelawangensis *	* timur *
Supralabials	9–12	9–13	8–12	9 or 10	10 or 11	9–11	11	10–12	9–13	10–12	12–14	9–12	9–12	10–12	9–12	10–12
Infralabials	9–13	8–11	8 or 10	9 or 10	8–10	8–10	10	8–11	7–11	9–12	10–11	8–11	8–10	9–11	9–11	8–10
Tuberculation	P	P	P	P	W	P	W	P	P	W	W	W	W	W	P	W
Tubercles on ventral surface of forelimbs	No	No	No	No	No	No	No	No	Yes	No	No	No	No	No	Yes	No
Tubercles in gular region	No	No	No	No	No	No	No	No	Yes	No	No	No	No	No	No	No
Ventrolateral fold tuberculate	No	No	Yes	No	No	No	No	No	Yes	No	No	No	No	No	No	No
Paravertebral tubercles	37–45	31–42	36–44	31–34	36–41	33–43	31	34–38	34–49	40–57	35–36	39–48	34–44	30–50	36–42	38–43
Longitudinal rows of tubercles	22–30	21–26	22–25	18–23	22–25	22–26	24	23–27	19–27	20–29	20–22	19–24	21–25	20–24	23–25	21–24
Ventral scales	32–40	36–40	31–39	29–33	32 or 33	29–34	41	33–36	17–28	31–46	36–39	26–33	38–43	31–43	31–36	31–40
4^th^ toe lamellae	21–25	21–24	21–24	22–23	20–23	21–26	24	22–27	19–23	20–24	21–23	19–24	19–21	20–25	21–24	21–25
Femoroprecloacal pores	39–45	37–41	41–46	32–36	39–41	33–39	46	41–46	28–42	31–38	26–29	17–22	30	30–36	36	21 or 22
Precloacal groove in males	Deep	Deep	Deep	Shallow	Deep	Deep	Shallow	Deep	Deep	Deep	Deep	Deep	Deep	Deep	Deep	Deep
No. of body bands	3(1) or 4	3(1) or 4	4	6 or 7	4 or 5	4	4	3	3–4	4	4	4	4 or 5	4 or 5	4	4
Body band/interspace ratio	1.00–2.00	1.00–1.25	1.00–1.25	0.82–1.10	0.50–1.25	0.75–1.25	1.75	2.00–2.75	0.95–1.74	1.00–2.00	0.75	1.00–1.25	0.75–1.00	1.00–2.00	1.00–1.50	1.00–1.25
Dorsum bearing scattered pattern of white tubercles	No	No	No	No	No	No	No	No	No	Yes	Yes	No	No	No	No	No
Hatchlings/juveniles with white tail tip	No	No	No	Yes	/	No	/	No	No	Yes	Yes	Yes	Yes	Yes	Yes	No
Adult posterior caudal region white	No	No	No	Yes	No	No	No	No	No	No	No	Yes	No	No	No	No
Dark caudal bands on original tail	7–8	8–10	8 or 9	9–11	14	8–10	7	6–7	7–10	13 or 14	>7	8–10	11–16	12–14	10	8–10
White caudal bands in adults immaculate	Yes	Yes	Yes	No	Yes	Yes	Yes	Yes	No	No	No	Yes	No	No	No	Yes
Maximum SVL	120.10	111.1	114.40	96.00	103.1	114.1	100.1	122.2	117.9	108.3	99.00	102.7	99.8	103.5	119.8	120.5
Sample size	12	14	6	3	4	13	1	6	39	11	3	14	10	13	4	5

Based on molecular data, *C. macrotuberculatus* is the sister lineage to a clade composed of *C. pulchellus* and *C. evanquahi*. It can be separated from those two species by having tubercles on ventral surface of forelimbs, gular region, and in ventrolateral body folds (*vs.* absent in *C. evanquahi* and *C. pulchellus*); 17–28 ventral scales (*vs.* 29–33 in *C. evanquahi* and 29–34 in *C. pulchellus*); deep precloacal groove in males (*vs.* a shallow in *C. evanquahi*); three or four dark dorsal bands (*vs.* six or seven bands in *C. evanquahi* and only four bands in *C. pulchellus*); white posterior caudal region absent (*vs.* present in *C. evanquahi*); hatchlings and juveniles without white tail tip (*vs.* present in *C. evanquahi*).

## Discussion

*Cyrtodactylus macrotuberculatus* and *C. phuketensis* are considered to be conspecific with the latter restricted to Phuket Island whereas *C. macrotuberculatus* is found on the Thai-Malay Peninsula and adjacent islands. The distinct characteristics between these two species were based solely on morphological comparisons by Sumontha et al. (2012). Our study provided additional morphology and molecular evidence to reassess the taxonomic status of *C. macrotuberculatus* and *C. phuketensis* from Thai populations and determine that these closely related populations are conspecific. Phylogenetic analyses from this study are concordant with the phylogenetic studies of Grismer et al. (2012, 2014, 2016), Quah et al. (2019), and Wood et al. (2020). Based on the dataset of ND2 gene and its flanking tRNA, the phylogenetic analyses recovered a clade of *C. macrotuberculatus* – including *C. phuketensis* – as a strongly supported monophyletic group consisting of multiple insular populations. Some substructuring occurs within the *C. macrotuberculatus* which could be the result of limited gene flow among isolated populations (Hurston et al. 2009; Jang et al. 2011) or local adaptation to different selection pressures in widely distributed habitat generalist.

Sumontha et al. (2012) diagnosed *C. phuketensis* by the number of bands between the limb insertions and the presence of a precloacal groove in the female paratype. We re-examined the type series of both species (except female paratype of *C. phuketensis*, QSMI 1170) and newly collected specimens. Variation in the number of bands was found in both species, similar to several species of the *C. pulchellus* group such as *C. bintangrendah*, *C. australotitiwangsaensis* and *C. lenggongensis* (Grismer et al. 2012, 2016).

Within the *C. pulchellus* group, a continuous series of enlarged femoroprecloacal scales forming an inverted T in the precloacal region is present in both sexes; however, the precloacal groove was found only in males. In the present study, the newly collected female specimens from the type locality of *C. phuketensis* had a continuous series of enlarged femoroprecloacal scales but lacked a precloacal groove (or depression) (Fig. 9). Therefore, this character is the same as in *C. macrotuberculatus* and all other species in the *C. pulchellus* group. The presence of a precloacal groove in the female specimen of *C. phuketensis* examined in Sumontha et al. (2012) was an erroneous observation (fig. 4 in Sumontha et al. 2012). The absence of a precloacal depression was used as a diagnostic character separating *C. macrotuberculatus* from *C. phuketensis* (see Grismer and Ahmad 2008; Sumontha et al. 2012). Based on the terminology of the precloacal depression in Mecke et al. (2016), the described specimens were re-examined and the presence of a precloacal depression (as precloacal groove) was observed in both *C. macrotuberculatus* (deep depression) and *C. phuketensis* (shallow depression). The PSUZC-RT 2010.58 and THNHM 15378 specimens are two males of *C. phuketensis*, in which the precloacal grooves are shallow (all others are deep) and could result from their poor state of preservation; thus, the character of this specimen was not included in the present diagnostic characters of *C. macrotuberculatus*.

Evidence from both overlapping ranges of morphology and relatively low sequence divergence indicate that *C. phuketensis* is an inconsistent pattern variation of *C. macrotuberculatus*. We concluded that *C. phuketensis* should be treated as a junior synonym of *C. macrotuberculatus* based on the priority of names designated by International Code of Zoological Nomenclature (ICZN). Additional surveys should be conducted to determine their geographic distribution and the degree of variation and patterns of gene flow within this species.

## Supplementary Material

XML Treatment for Cyrtodactylus macrotuberculatus
